# No effect of different types of media on well-being

**DOI:** 10.1038/s41598-021-03218-7

**Published:** 2022-01-06

**Authors:** Niklas Johannes, Tobias Dienlin, Hasan Bakhshi, Andrew K. Przybylski

**Affiliations:** 1 Oxford Internet Institute, University of Oxford, Oxford, UK; 2 University of Vienna, Vienna, Austria; 3 Creative Industries Policy and Evidence Centre (PEC), Nesta, London, UK

**Keywords:** Psychology, Human behaviour

## Abstract

It is often assumed that traditional forms of media such as books enhance well-being, whereas new media do not. However, we lack evidence for such claims and media research is mainly focused on how much time people spend with a medium, but not whether someone used a medium or not. We explored the effect of media use during one week on well-being at the end of the week, differentiating time spent with a medium and use versus nonuse, over a wide range of different media types: music, TV, films, video games, (e-)books, (digital) magazines, and audiobooks. Results from a six-week longitudinal study representative of the UK population 16 years and older (N = 2159) showed that effects were generally small; between-person relations but rarely within-person effects; mostly for use versus nonuse and not time spent with a medium; and on affective well-being, not life satisfaction.

## Introduction

Does media use have observable effects on well-being? Historically, the public and scholars have been skeptical of new media and technologies^1^. For example, there is a lively debate on the harmful effects of digital devices (e.g., smartphones) or new media (e.g., social networking sites)^2–5^. The lockdown during the COVID-19 pandemic reignited that narrative of overreliance on technology, because people were using media much more than previously^6,7^. That narrative is ambivalent: Technology use is considered an enjoyable pastime and simultaneously harmful. Additionally, this discourse places high value on ‘traditional’ media like books, but considers ‘newer’ media, such as social networking sites, of low value^1^. The literature reflects that discourse; most work investigates effects of newer media^8–10^. Highlighting the alleged benefits of traditional media without a comparative evidence base of their effects on well-being runs the risk of perpetuating an elitist view of media use^1^. It also fails to deliver actionable evidence to policymakers who need to decide whether to encourage or discourage media use^11,12^. Here, we present such evidence.

Media effects can occur on different levels^13^. When asking about the effect of using a medium on well-being, we typically begin on the most general level to study the ‘net’ effect of a behavior^11^. In the case of new media, this general level is represented by screen time^10,14,15^. Subsequently, researchers have studied new media with increasing nuance. They have come to understand that the motivation of users is just as important as the content they engage with^3,5,14,16^. For example, users might compare themselves to others on social media; depending on their motivation, that comparison can make them feel better or worse^17^. This level of nuance is critical. However, it must build upon a robust understanding of broad, net effects. An investigation of this broad effect is lacking for more traditional media. Consequently, we cannot compare net effects of social media to those of traditional media. That comparison is important because societal discourse often takes a form of technological determinism^12^ for granted: People are helpless in the face of social media and attention demanding notifications, but benefit from traditional media, such as reading books^1^. Therefore, our first research question asked about the effects of media use on well-being over a broad range of traditional media.

Dominated for a long time by cross-sectional work, recent longitudinal studies have begun adding nuance to the analysis of net screen time effects^15,18^. For one, they distinguish within-person and between-person relations. Between-person relations represent stable differences, but not effects; within-person relations *can* represent effects^19^, depending on whether there are time-varying confounders^20,21^. Therefore, several scholars have recommended applying this distinction to the study of media effects^16,22^. Second, researchers routinely acknowledge that it is unlikely that screen time affects well-being, but not vice versa. Therefore, most work now models reciprocal relations. Between-person relations between screen time and well-being are negative, but small, with those reporting higher technology use also showing slightly lower well-being^8,9,23,24^. On the within-person level, effects are negligible: If a person uses technology more than they usually do, any effects on well-being are too small to matter practically^8,9,25–28^. These negligible effects appear to be reciprocal^8,9,29^. Unfortunately, we do not have such insights for traditional media. Therefore, our second research question asked about the reciprocal effects between media use and well-being, distinguishing between-person relations from potential within-person effects.

Technological determinism also contributes to a focus on researching only how much a person uses technology, but rarely whether someone uses the technology in the first place. An implicit linear dose–response model underlies this focus: Research asks how much technology will yield what amount of harm^30,31^. But the decision whether to use a medium or not is most likely a different psychological process than the sheer amount of time people use that medium for. Deciding to read a book might be good for our well-being, regardless of whether we read for ten minutes or two hours. Conversely, it might not matter whether we pick up a book if we only spend a few minutes reading. So far, research has treated the step from zero to one minute as similar to the step from 100 to 101 min, even though use versus nonuse likely has larger consequences than merely spending a minute more with a medium we already use a lot. The few studies looking at users versus nonusers are in a similar state as the research on screen time five years ago. Although they reveal some interesting differences, they are mostly cross-sectional^32,33^. Whereas researchers study time spent with media with increasing nuance by employing longitudinal designs and separating between- and within-person levels, we lack that nuance for use versus nonuse. We are aware of only one study that reports a small positive within-person association between using versus not using social media and well-being^34^. Therefore, our third research question asked about the different effect of use versus nonuse and time spent with a medium.

The question of how much screen time will harm people has also overshadowed another important question: On what facet of well-being should we expect effects and how long does it take for media use to affect well-being? Most research has relied on relatively broad and stable indicators of well-being, a component of mental health, that address evaluative, cognitive well-being, such as life satisfaction. Because life satisfaction is remarkably stable over longer periods of time, it is questionable whether media use can produce changes strong enough to impact how people evaluate their lives^16^. Cohort studies with yearly lags^9,28,35^ or lags of several months^36,37^ have produced mostly null findings on the within-person level. Instead, researchers have argued that media use should impact the affective component of well-being, such as the positive or negative affect that people experience^16,38^.

The question of what type of well-being media use influences is therefore closely tied to the lag in measurement. If media use indeed has an effect on well-being, we should observe it in the short-term and on affect. Life satisfaction might be too stable—or the net influence of media use too small—to change in the short-term; alternatively, media effects might be too weak to change life satisfaction even in the long-term. For example, there is strong evidence that social media, internet, and TV use do not impact life satisfaction to a meaningful degree over several years^9,27^. On the flip side, even recent studies using (half-) hourly lags found no meaningful effect of social media use on affect^34,38,39^. Such short lags are in line with recommendations to use more “shortitudinal” studies^40^, but the net effect of new media might still be too small to impact affect immediately. Instead, small media effects may accumulate in intermediate time frames, such that we experience an effect on well-being only after enough use episodes^41^. For example, binging TV shows over a week may only have small, fleeting effects right after watching, but could accumulate and fully unfold its effects by the end of the week. The data set we analyzed allowed us to test this proposition: It had a relatively short time lag of one week and measured both affect and life satisfaction. Therefore, our fourth research question asked about the effect of media use on different indicators of well-being in a relatively short time span.

### The current study

We explored the question of the effects of diverse forms of media use on well-being and vice versa by analyzing an existing data set of a nationally representative cohort of people living in the UK over six weekly measurements during the first national lockdown (April and May 2020). We had four research questions: First, we tested the effects of a wider range of *seven more traditional media*: music, TV, films, video games, (e-)books, (digital) magazines, and audiobooks. Second, we separated *between-person relations* from *within-person effects.* Third, we analyzed both *use *versus* nonuse* and *time spent using a medium*. Fourth, to get a better understanding of which well-being concepts media use most likely affects, we *compared effects* on both affect (i.e., more volatile indicator) and life satisfaction (i.e., more stable indicator). Together, these steps deliver a comprehensive base of evidence over a range of traditional media that is lacking from the literature.

We did not preregister a processing and analysis plan for secondary data^42^. Instead, we followed calls for more transparency in the scientific process^43,44^ and share all data, materials, and code on the Open Science Framework page of this project (https://osf.io/yn7sx/). Readers can find detailed documentation of data processing and analysis at https://digital-wellbeing.github.io/cultural-consumption/ (further referred to as online supplementary materials).

## Results

**Figure 1:**
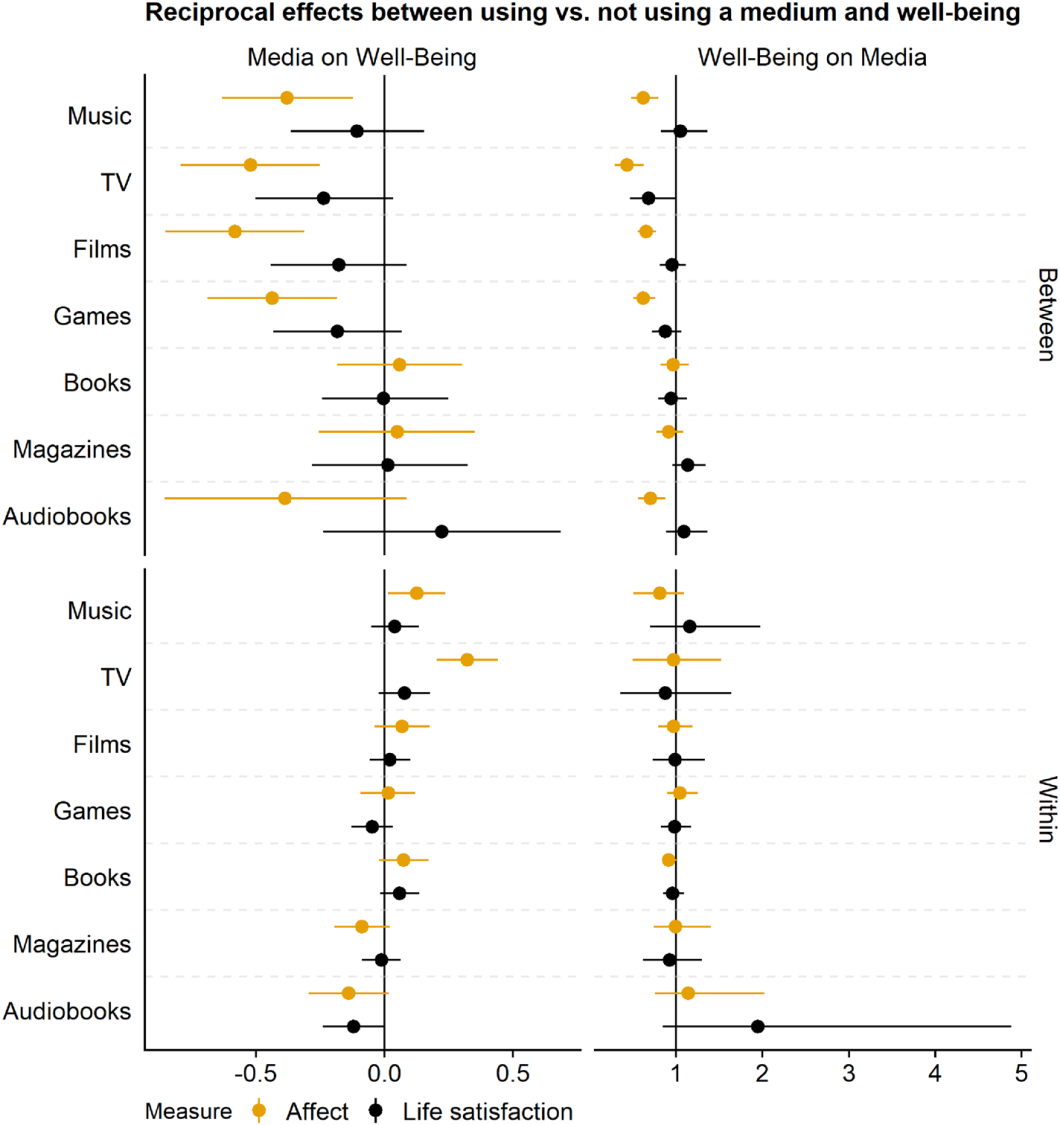
This Figure shows the reciprocal effects between using versus not using a medium and well-being. Points represent the mean of the posterior distribution of the effects. Lines represent 95% Credible Intervals. On the left-hand column, coefficients are of the Gaussian models; they represent an unstandardized estimate on the 0 to 10 well-being scale. On the right, coefficients are of the hurdle gamma model, meaning they represent odds of using a medium at all (hurdle part) and time spent using a medium more (gamma part). The top part shows between-person relations. The bottom part shows within-person effects. Estimates are grouped by the well-being measures, affect versus life satisfaction.

**Figure 2:**
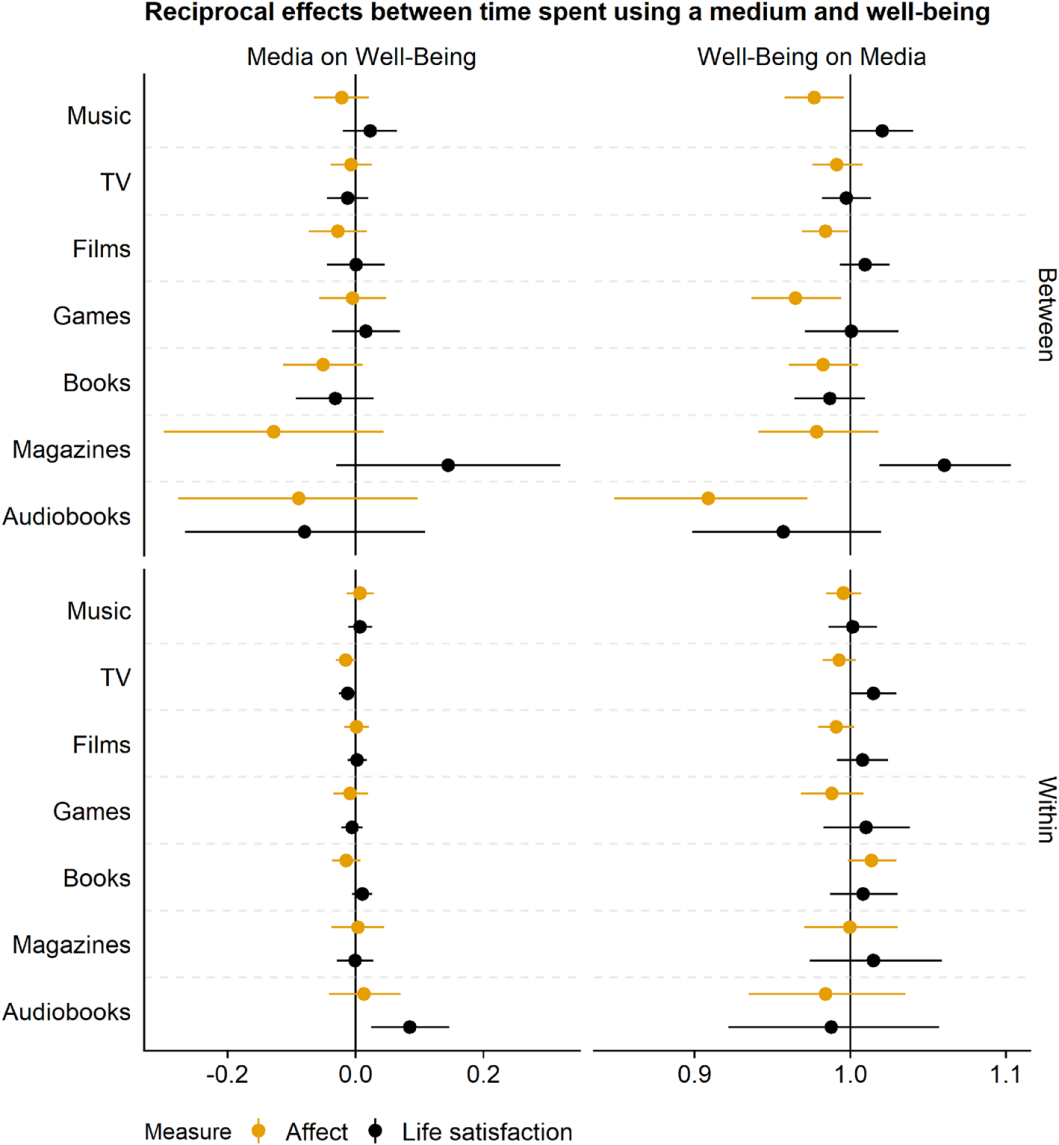
This Figure shows the reciprocal effects between time spent using a medium and well-being. Points represent the mean of the posterior distribution of the effects. Lines represent 95% Credible Intervals. On the left-hand column, coefficients are of the Gaussian models; they represent an unstandardized estimate on the 0 to 10 well-being scale. On the right, coefficients are of the hurdle gamma model, meaning they represent odds of using a medium at all (hurdle part) and time spent using a medium more (gamma part). The top part shows between-person relations. The bottom part shows within-person effects. Estimates are grouped by the well-being measures, affect versus life satisfaction. Note that the range on the x-axis is different from Fig. 1 to show the CIs.

The analyses testing our research questions showed that estimates we observed were generally small; between-person relations but rarely within-person effect; mostly for use versus nonuse and not time spent with a medium; and for affect, not life satisfaction. Figures 1 and 2 present an overview of the results. For the exact numerical coefficients, see the online supplementary materials: https://digital-wellbeing.github.io/cultural-consumption/synthesis.html#full-table-of-estimates. Note that both facets in the top-row in both figures represent the same theoretical effects. However, they were obtained from different models, explaining their small differences.

### Example: music

Before we turn to the research questions, we provide an example that (a) showcases how to interpret the model estimates, and (b) demonstrates the complexity of media effects. In Fig. 1, in the top left, we see the between-person relation between listening to music versus not listening to music and affect and life satisfaction. Across the entire sample and all waves, the model estimated that someone who listens to music, compared to someone who does not listen to music, reports feeling somewhat worse on affect, but not on life satisfaction (on the raw 0 to 10 scale). That correlation is mirrored on the top right that shows relations between affect and life satisfaction, across all waves, and whether someone listens to music versus not. Mirroring the left-hand columns, someone who feels one point better than someone else on the 0 to 10 affect measure has lower odds of listening to music compared to someone else. More importantly, the bottom left shows within-person effects of listening to music—the most relevant outcome of our models because they inform us about media effects rather than between-person correlations. Going from not listening to music to listening to music makes a person feel slightly better by the end of the week. Conversely (on the bottom right), if a person feels one point better on affect than they typically do at the beginning of the week, their increase in affect does not increase their odds of listening to music in that week.

In Fig. 2, we see the estimates of time spent listening to music. The top left corner shows that one hour more music listening than someone else is only weakly related to lower well-being (affect); conversely, scoring one point higher on well-being (affect) than someone else is only weakly related to lower odds of spending more time listening to music. At the within-person level (bottom left), when a person listens to one hour more than they typically do, their listening makes them feel slightly better, but the posterior included zero as well as negative effects. Likewise, if someone scores one point higher on the affect scale than they typically do, their increase in affect does not increase their odds of listening to more music (bottom right).

### Differences across media

Addressing our first research question, between-person differences of users versus non-users were comparable across all media—mostly in direction of the difference, but also in size. People engaging with music, TV, films, and video games felt generally worse than those who did not engage with those media. Those reading books or magazines or listening to audiobooks did not feel better or worse than those who did not. Again, these differences represent correlations. It is just as possible that people who feel bad generally turn to music, TV, films, and video games, but it matters little how a person feels when they choose to read books, magazines, or listen to audiobooks. On the within-level, differences between media were even smaller: Increasing media use above a person’s typical use, whether using a new medium or engaging for longer with a medium, had close to no effect on their well-being, regardless of the medium. Even effects whose posterior distribution excluded zero, as was the case for TV and music use versus nonuse, were small. The same goes for the other direction of the effect: Feeling better than usual did rarely lead to a change in the odds of picking up a new medium or engaging more with an old one. That lack of a change applied across all of the media types.

### Between versus within

Results concerning our second research question show that most relevant estimates happen on the between-person level. These differences represent stable differences between people across all waves. They represent correlations and not causal effects. They show mostly small negative relations between media use and well-being. More specifically, these relations appear to be driven by differences between using versus not using a medium, not by how much time people spend with that medium. Those people who use a medium, compared to people who do not use the medium, in general feel worse. Again, this conclusion is not causal, and it works both ways: In general, those who feel worse than others have higher odds of using a medium. More importantly, on the within-level, very few posterior distributions excluded a null effect. Those that did represented small effects (e.g., TV and music).

### Use versus time spent

To test our third research question, we inspected general differences between using versus not using a medium and time spent with a medium. We see that most effects whose posterior excludes zero occur for whether a person uses a medium and less so by how much time they spend engaging with that medium. These effects are also generally larger and less certain than effects of the time spent with a medium. Most coefficients describing effects on and of time spent with a medium are extremely small. Even if their posterior excludes zero, we can be relatively certain—conditional on the model and the data generating process we assumed—that the true underlying effect is small to negligible. For example, going from not watching TV to watching TV results in about a third of a point increase by the end of the week on the eleven-point affect scale. But one hour more TV time than a person typically watches results in a hundredths of a point decrease by the end of the week, and the posterior of that effect includes zero.

### Affect or life satisfaction

Addressing our fourth research question, almost all differences we observed on the between-person level were in affect, not in life satisfaction. In other words, users and nonusers of media differ on how anxious and happy they feel across the study period of six weeks (i.e., the items to measure affect). They differed less on the degree to which they evaluate their lives in general. Even though most posterior distributions of the between-person differences on life-satisfaction were negative, they all included the null effect as a plausible value that represented the true underlying difference (conditional on the model). Similarly, the few within-person effects whose posterior excluded the null effect were on affect. For example, those who picked up watching TV reported higher affect by the end of the week, but not higher life satisfaction.

## Discussion

New media like social networking sites allegedly exert an almost addictive effect on their users, whereas traditional media like books are considered a beneficial pastime. However, the alleged benefits of traditional media remain speculative without much evidence of their effects on well-being. We set out to deliver initial evidence of the broad, ‘net’ effect of a range of traditional media. First, we investigated media effects across a wide range of *seven traditional media*. Second, in a reciprocal analysis we separated *within-person effects* from *between-person relations*. Third, we treated *use *versus* nonuse* and *time spent with a medium* as different processes. Last, we analyzed data with a shorter time lag than most previous work, testing which *facets of well-being* are affected most by media use. Our findings provide little cause for alarm: Almost all differences were between users and nonusers on a stable between-person level, with small to negligible within-person effects in either direction. The few effects we found were comparable across media and largely on the (more volatile) affective well-being, rather than more stable life satisfaction.

Distinguishing use versus nonuse and time spent with a medium proved important. Most differences we observed were on the between-person level between users and nonusers. Likewise, the few small within-person effects incompatible with zero as the true effect occurred when a person went from not using a medium in one week to using a medium the next week. The time spent with a medium played a negligible role. In other words, our findings are not in line with the dominant linear dose–response model that (often implicitly) assumes that going from zero use to one minute of use has the same effect as going from one hour of use to one hour and one minute of use^30,31^. Instead, the decision to use a medium appears to represent a threshold; once a user crosses that threshold, the amount of time they spend with a medium is of little consequence for their well-being.

This conclusion almost exclusively applies to the between-person level: Media users (i.e., those who have crossed the threshold) in general feel slightly worse than nonusers (i.e., those who have not crossed the threshold). However, those differences were around a third of a point on an eleven-point scale. Such an effect is likely too small have practical significance for people’s lived experience^45,46,47^. On the within-person level, going from nonuse to use had generally small effects across media. The effects of time spent with a medium were even smaller. Our results speak against pronounced causal effects—neither positive nor negative—of media use during the week on well-being by the end of the week. The pattern of small between-person relations but negligible within-person effects aligns with previous research on new media^8,9,23^.

There were no substantial differences across the seven traditional media types we studied. (E-) book and (digital) magazine readers as well as audiobooks listeners did not experience less affective well-being unlike those engaging with music, TV, films, and games. That finding applies in both directions: Those with lower well-being were more likely to engage with these media. However, those differences all but disappeared on the within-person level, with most effect sizes close to a null effect. Only TV and music use versus nonuse on the within-level showed a small positive effect on affect. Together, the results stand in contrast to public opinion, where traditional media are valued highly^1,48^. It appears the broad, net effect of traditional media is similar to that of social media: too close to zero to be perceived by media users^45^.

Our study also addresses the choice of time lag and well-being indicator. Media effects are typically small^49^ and it is unlikely that media use will affect long-term evaluations of people’s lives^16^. If anything, media use should influence short-term affect. Our results deliver weak evidence that this distinction also applies to traditional media. The few differences we observed appeared almost exclusively on the more volatile positive affect, not stable life satisfaction. These results align well with research that shows little to no long-term effects of new media on life satisfaction^9,27,28,35^. We deliver evidence that traditional media are unlikely to impact life satisfaction within the intermediate time frame of one week that we studied. At the same time, the few effects on affect were small, similar to research on social media with much shorter time lags^34,38,39^. Either we missed the optimal time lag after which the effects disappeared^40^ or net effects of traditional media are indeed negligible.

What do our results mean? The straightforward answer is: The effect of traditional media on well-being is too small to matter. However, such an answer might overlook important nuance. First, throughout this manuscript, we have spoken of between-person relations, but of within-person effects. As we have noted, within-person relations can be effects under the assumption that there are no time-varying confounders. Therefore, what we call effects is causal only under that assumption^20,21,50^. There might well be time-varying factors that mask a true effect^51^. For example, spending time using media may have a negative effect on well-being which gets balanced out by an indirect positive effect via less time worrying. Similarly, a stable confounder (e.g., employment status) might drive the small negative between-person relation. Alternatively, people who do not feel well might indeed be more inclined to pick up a new medium as a mood management strategy^52^.

Second, we only investigated the broad, net effect of traditional media. We did not assess what content people engaged with or what their motivation for use was. Although we believe such net effects are important to investigate as first step, they may mask important interactions between content and user motivations^31,48,53^. Therefore, even though within-effects of traditional media are small, there may be meaningful under certain conditions^54^. Such an argument aligns with research which found noteworthy variation in the effect of social media^34^. Third, we looked at an intermediate time lag of one week, which might have missed the effect. Therefore, to revise the answer from above: *Under our assumptions of causality*, the *broad, net* effect of traditional media *during the week* on well-being *at the end of the week* is likely too small to matter.

### Limitations

Besides the questions of causality and scope of media use, there are several limitations to our study. The self-reported estimates of time spent with a medium we relied on will be almost certainly a noisy measure of true media engagement^55,56^. In addition to that noise, the measures also reminded participants of their response in the previous week. That reminder might have reduced variance or introduced bias. By contrast, we believe self-reports of use versus nonuse in a one-week period have lower measurement error, simply because there are more biases in retrieving exact estimates of the behavior compared to a dichotomous yes/no retrieval. We call for more research directly measuring media use. Similarly, although they displayed decent psychometric properties, the well-being measures in the data set were not validated. The measure of affect in particular referred to affective well-being on the previous day, not the previous week. Although it allows a sensible test of the cumulative effect of media use during the week on well-being at the end of that week, the opposite direction is less plausible: Affect at the beginning of the week might not be strong enough to influence media use during the week that follows. Most important, we did not assess social media use, which prevents us from a direct comparison of the effects of traditional media versus new media. Although our results fit into the larger picture of the literature, a direct comparison will be more informative.

### Conclusion

Our results do not suggest that using traditional media harms or benefits users in the intermediate; an elitist view of, for example, books does not seem warranted. Based on these broad, net effects there seems to be no need for policymakers to encourage or discourage media use on the basis of well-being alone. These results only represent a first step. There is much to learn on the optimal time lag, underlying causal models, potential confounders, and what people actually do with media, rather than relying on a simplistic linear dose–response model. The question of whether media use is *generally* bad has been answered with a convincing no in the past five years. It is time to collect *fine-grained* data that combine various time lags with objective measures of different types of behavior, content, and motivations for interacting with media. Such data would enable societal and scholarly discourse to move on to more pressing questions about the role of media in our lives.

## Method

### Sample and procedure

We analyzed data from a nationally representative sample of the UK. The data were part of a larger panel study undertaken by the Creative Industries Policy & Evidence Centre, led by Nesta, an innovation foundation (https://www.nesta.org.uk/) and the Intellectual Property Office (IPO). The project was commissioned to monitor the impact of the COVID-19 pandemic on how people’s cultural consumption changed during lockdown and thereby impacted the creative industries (https://www.pec.ac.uk/assets/publications/PEC-and-IPO-cultural-consumption-study-wave-8.pdf).

Data collection took place between 9th of April and 24th of May 2020 and was managed by the research agency AudienceNet. AudienceNet distributed six weekly surveys to one of their online UK consumer research panels that is representative of the UK population aged 16 and older. Quotas were set, using data from the Office for National Statistics, for age/gender and Government Office Region/Devolved National Administration.

Data collection followed regulations of the Intellectual Property Office on questionnaire surveys and interviews. As a government agency, the Intellectual Property Office does not require approval from a dedicated ethics committee for collecting the types of data analyzed here. Employees from both the IPO and Nesta reviewed the ethics procedure of AudienceNet, the research agency that carried out data collection. AudienceNet is an accredited Company Partner of the Market Research Society (MRS) and followed MRS guidelines and codes of conduct. Therefore, the study protocol was carried out in accordance with relevant guidelines and regulations. Respondents who were recruited for the survey had previously given informed consent to take part in a research panel. Before starting the survey, respondents read information about the types of questions they would be asked to complete. That information explained that their data would be anonymous and that they could withdraw from the study at any time. Choosing to complete the survey beyond that point represented informed consent, which was obtained from all participants.

**Figure 3:**
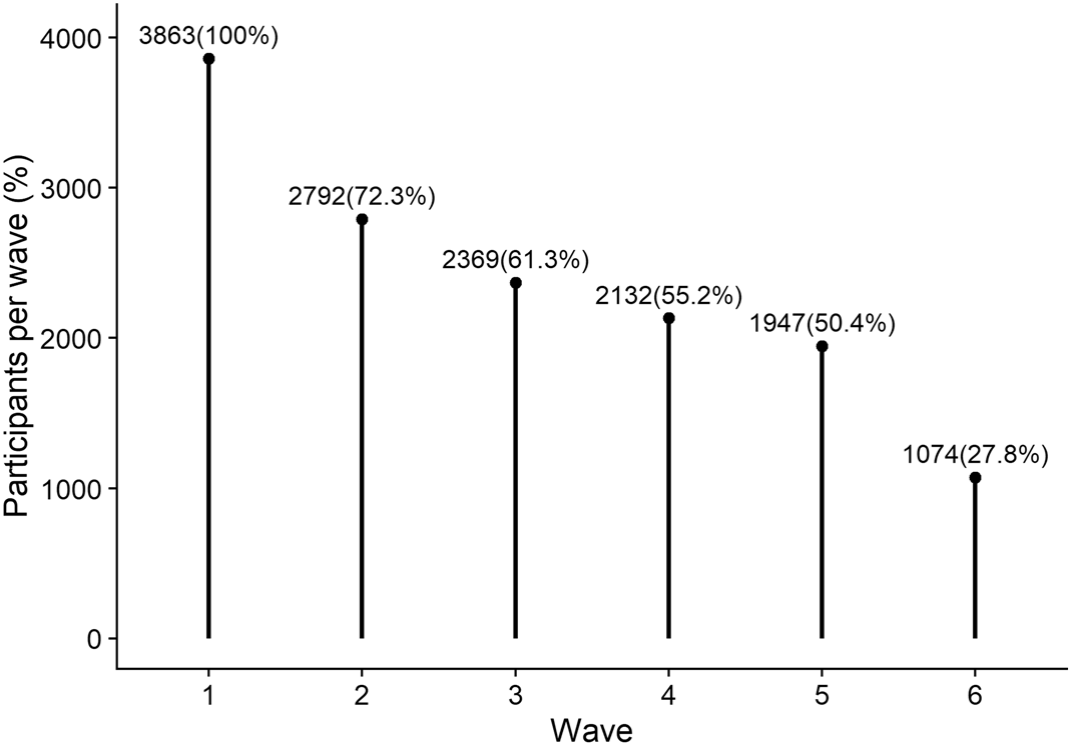
Participants who completed each wave. Participants were the same across waves and were only contacted if they completed the previous wave. In parentheses, we show the attrition rate.

At each wave, participants reported on their previous week (Monday to Sunday). The first survey took about ten minutes to complete; the following five surveys took between five and seven minutes. Participants received a fee for their participation which increased in later waves to disincentivize sample attrition. We show the sample size at the first wave and subsequent attrition in Fig. 3. We analyzed the data of respondents with at least three waves (see “Analysis” section for an explanation). For demographic information on participants who completed less or more waves, see https://digital-wellbeing.github.io/cultural-consumption/descriptives-and-visualizations.html#fig:relation-by-age.

**Figure 4:**
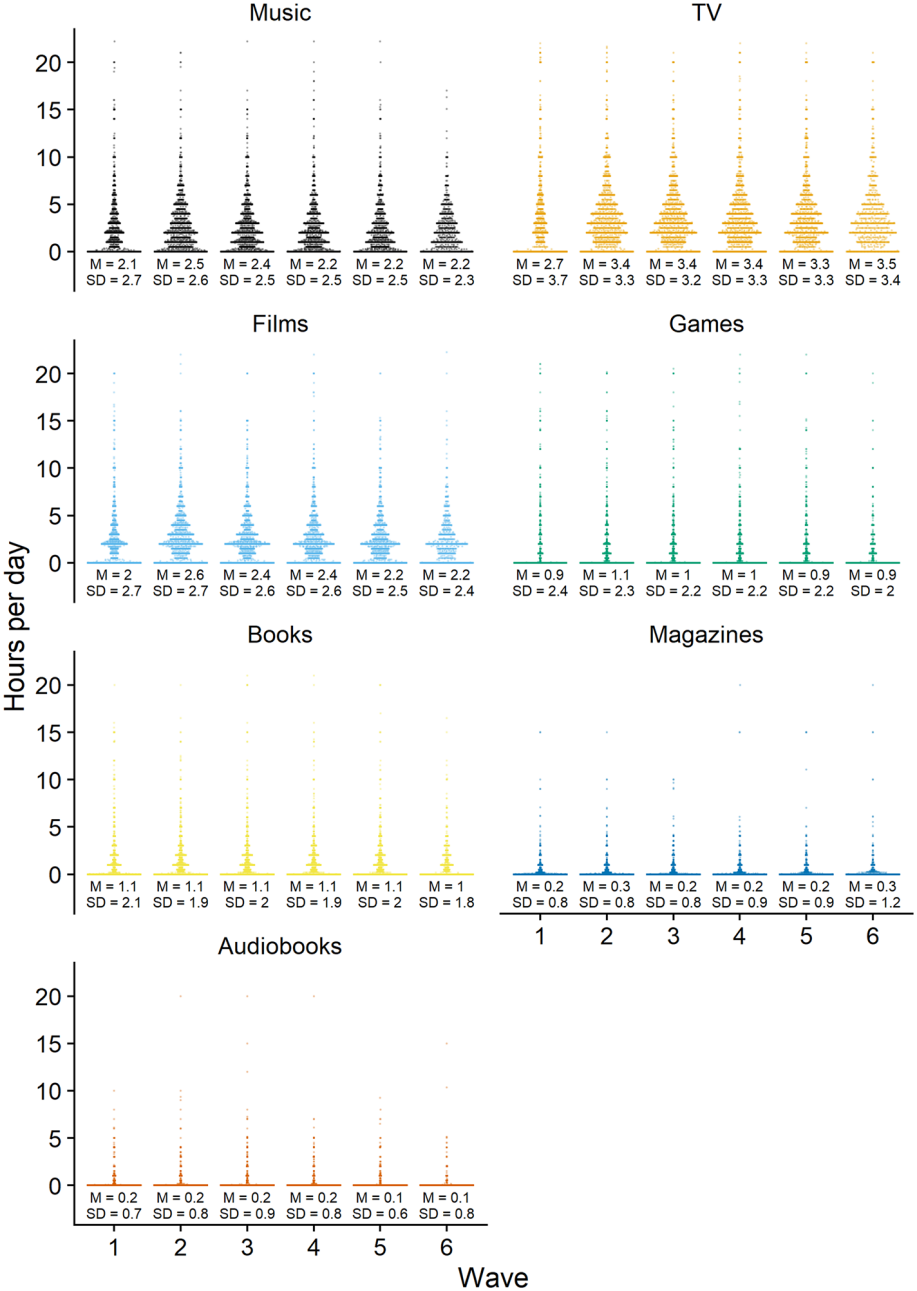
The figure shows the distributions of media use for each medium plus means and standard deviations.

At the first wave, participants provided demographic information in a first section. In a second section, participants reported whether they had downloaded, streamed or accessed, shared, or purchased physical copies of seven different media (see “Measures” and Fig. 4). If they had engaged with any of these media, participants provided detailed information on each medium in a third section. Among that information, they reported how much they identified with a certain medium and for how long they had used it in the previous week. We only used the degree of identification for exclusion criteria and the amount of time with a medium for the analysis; we did not examine nor analyze any other variables from this section. In a fourth and final section, participants reported on a variety of digital cultural activities and their well-being; we only used the well-being variables for analysis.

Waves two through six were identical to the wave one survey, except participants were not asked to report full demographic information or for how much they identified with a medium anymore. Even if they reported not having used a medium in the first wave, they were asked at each subsequent wave whether the amount of time with a medium had changed compared to the previous week. If they reported a change (rather than not having used the medium) they were again asked to provide an estimate of the time spent with the medium. We used those estimates of change for data processing, but not during the analysis (see “Measures” section).

#### Exclusions

Survey data often contains responses provided by participants who were not motivated or did not understand the questions as presented. Such responses can severely limit the accuracy of the inferences we can draw from the data. To ensure high data quality that allows valid inferences, it is recommended to exclude low-quality data prior to the analysis^57,58^. We followed recommendations to identify meaningless data by inspecting implausible values and response patterns, such as straight-lining^58^. We aimed to be as liberal as possible. That is, we wanted to avoid excluding extreme, but valid data and only identify responses that were clearly meaningless. We made those decisions before the analysis (https://github.com/digital-wellbeing/cultural-consumption/commit/358032c367c5c43f127459e9c17db39ac26dd223).

On the participant-level, we applied five exclusion criteria aimed at identifying participants who rushed through the survey or did not take it seriously. For example, we excluded everyone who had more than one instance of reporting to have used a medium for more than 23 h daily. On the wave-level, we had three exclusion criteria. For example, we excluded any wave where the sum of all daily use was above 64 h. Applying all exclusion criteria removed about 10% of observations and 9% of participants—both relatively low estimates of meaningless data^57,58^. Of the 2,159 remaining participants with data for at least three waves, 52% identified as women (*M*_*age*_ = 47, *SD*_*age*_ = 15). For a detailed account of the rationale behind exclusion criteria and their impact on the sample size, see https://digital-wellbeing.github.io/cultural-consumption/data-processing.html#data-quality-and-exclusions

### Measures

#### Identification

In the first wave, when participants reported having used a medium, they reported to what extent they identified with that medium (e.g., “I know more about music than most people I know”) on a Likert-type scale from 1 (*strongly agree*) to 4 (*strongly disagree*). We used these scales for the straight-lining exclusion criteria, but not for analysis. Full details are in the online supplementary materials.

#### Estimates of change

At the beginning of each wave, starting at wave two, participants reported on a scale from 1 (*increased a lot*) to 5 (*decreased a lot*) on the question “Compared to the week before, on average has the amount of time you have spent [engaging with a medium] each day changed?”. We only used those estimates to impute zeros for the media use estimates (see below). Details are in the online supplementary materials.

#### Media use

Participants reported their daily use of seven kinds of media: music, TV, films, video games, (e-)books, (digital) magazines, and audiobooks. For books, participants were asked about both physical and e-books. The same applies to magazines. In the first wave, for each medium, participants reported hours and minutes for the question “In the past week, on average how much time did you spend [engaging with the medium] each day?”. For each medium, the question had additional information. For example, the music question was followed by “This includes music you stream online (e.g. on Spotify or YouTube), music you have downloaded (e.g. in your iTunes library) and physical copies (e.g. CDs) you own.” From wave two onwards, the survey showed participants how much time they had reported in the previous wave with the reminder “Here is how much [medium] you said you [engaged with] on average each day when you completed the last survey a week ago”. Participants then proceeded to fill in their media use, prompted by the instruction: “Please type in the average amount of time you have spent [engaging with the medium] each day for the last week. If it has not changed, you can just type the same amount of time.”

We were not only interested in reciprocal effects of media use on well-being among active users, but also in the effects of using versus not using a medium. Therefore, whenever a participant reported not having used a medium, we treated their media use as zero. We imputed these zeros in two places. First, when participants in the first wave reported not having used a medium, we set their media use from missing to zero. Second, we treated missing values as zero in subsequent waves when (1) participants reported not having used a medium or (2) reported that their use remained the same after they had not used a medium in the previous wave. This decision to also analyze users versus non-users resulted in substantial zero inflation, in particular for media that respondents used sparingly (e.g., audiobooks). See Fig. 4 for a visualization of the distributions that show spikes at zero.

**Figure 5:**
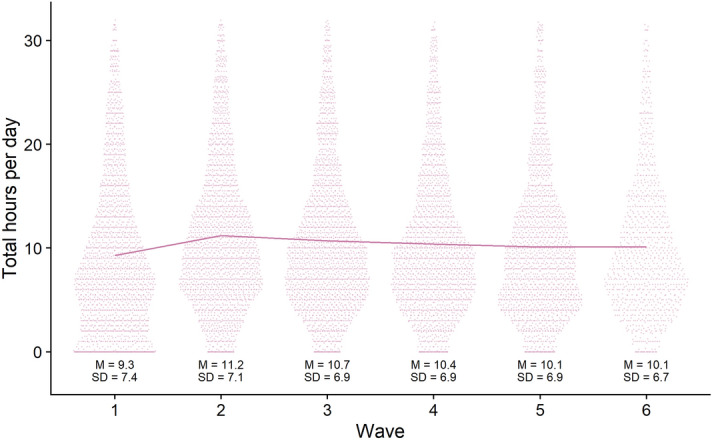
The figure displays the summed media use of all media plus means and standard deviations. The line connects the means between waves.

For description, we summed all media use to form total media use. Figure 5 shows three noteworthy trends. First, there was an increase in media use from week two (i.e., third week of April 2020). Second, besides this early bump, average use for all media remained stable, only slightly decreasing over the remainder of the study. Third, respondents were not good estimators of media use. Their daily total media use, on average, took up almost half of each day—mostly likely an overestimate^48,56,59^. A substantial number of respondents reported daily media use of more than 20 h. We take this pattern as evidence that we can analyze relative changes in self-reported media use, but we cannot treat these results as a descriptive measure of absolute media use.

**Figure 6:**
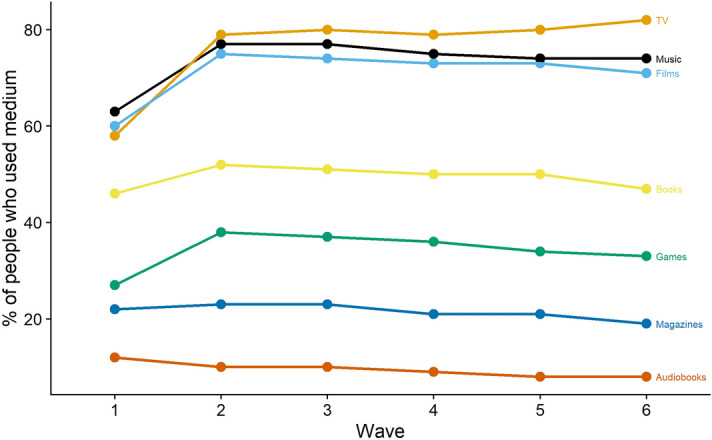
The proportion of respondents who used a medium at each wave. Note that it is the proportion of the final sample that we analyzed, so after exclusions and only those with at least three waves of data.

We also plotted the proportion of users who used a medium at each wave. Figure 6 shows that, analogous to the increase in total time at wave 2, more people started using media after the first week. That proportion remained stable in the following waves.

#### Well-being

**Figure 7:**
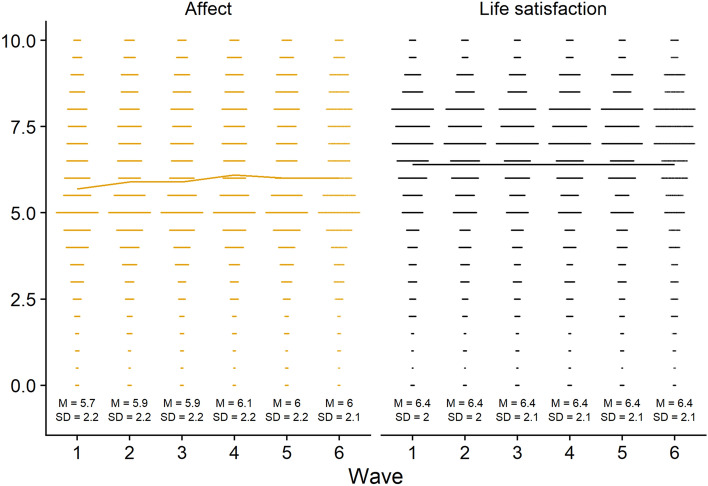
The figure shows the distributions of both well-being measures plus means and standard deviations. Lines connect the means between waves.

At the end of each survey, participants answered two questions about their satisfaction with life and two questions about their affective well-being (from here on “affect”). Life satisfaction items were “All things considered, how satisfied are you with your life as a whole nowadays?” and “To what extent do you feel that the things you do in your life are worthwhile?”. Affect items were “How happy did you feel yesterday?” and “How anxious did you feel yesterday?”. Respondents rated their well-being on an eleven-point scale, ranging from 0 (*not at all*) to 10 (*completely*). For both concepts, we took the mean of the two items (reverse coding the anxiety question). Naturally, both measures were related, but not strongly enough to treat them as a unitary concept (*r* = 0.65). For an analysis of their factor structure, see the online supplementary materials. Figure 7 shows the distributions and means of both measures. Both affect and life satisfaction remained stable over the duration of the study (i.e., the largest increase in affect was only 0.4 points on an 11-point scale).

### Analysis

We processed and analyzed data with R^60^. For data analysis, our models had to accommodate our research questions as well as the properties of the data. First, we were interested in lagged, reciprocal effects of media use on well-being. Second, following previous research, we distinguished between-person relations and within-person effects^9,19^. Third, we differentiated between effects of/on use versus non-use and media use (i.e., time with a medium). Fourth, models addressed that media use was not normally distributed, but had zero inflation.

We relied on a mixed-effects model approach as it can accommodate all of these demands. Specifically, we ran so-called Bayesian random effect within between models^61^. To address lagged, reciprocal effects, we ran two types of models, one for each direction of effect. Models predicting well-being took into account that the data were naturally lagged: Respondents reported their *current* well-being but their media use during the *previous week*. Therefore, we ran Gaussian models predicting current well-being from media use in the previous week. In effect, these models predicted how participants felt at the end of the week with their media use during the week, following our rationale that media use effects might cumulate over the duration of the week. Models predicting media use reversed that logic: We ran a hurdle gamma model that predicted media use during the week with well-being at the beginning of that week. We needed at least two measures per person and because the later model used a lagged predictor, we included only participants with at least three waves of data. Relying on such an approach had the additional advantage that we did not need to restrict ourselves to participants with data on all six waves: The models weigh responses from participants with more data more heavily, but do not discard participants with less data.

To differentiate between-person relations from within-person effects, we cluster-mean centered predictors and used the cluster means (as between-person association) and each participant’s deviation from their cluster mean (as the within-person effect) to predict the outcome^62^. Consequently, there were two predictors for each well-being variable (cluster mean and deviation). A model estimate of the between-person association tells us the difference in media use for someone with well-being one point higher than someone else. A model estimate of the within-person effect tells us the effect of going up one point from that person’s typical well-being.

For media use, there were four predictors, two for use versus nonuse and two for time spent with media (i.e., time when a medium was used). For use versus nonuse, the cluster (i.e., person) mean represented the proportion of waves that a participant used a medium across all waves. Because the proportion was bounded at [0,1], a one-point increase estimated by the model represents the difference between a non-user (proportion of zero) and a user (proportion of 1). The within-person effect was again represented by a deviation from that cluster mean (so cluster mean minus zero if a person had not used a medium at a certain wave or minus one if they had). Again, because the predictor was bounded, a model estimate of an increase of one of the within-person effect represented the effect of going from not using a medium in one wave to using a medium in the next wave. Separating use from time also addressed our third goal to distinguish between these two processes for models predicting well-being.

To account for the non-normally distributed media use and to separate effects of well-being on media use, we predicted media use with hurdle gamma models. We assumed that media use followed such a two-part data generating process, where zero versus one is a hurdle of whether someone used a medium (first part), and, if they used a medium, that time was not normally distributed (second part). Well-being predicted both the hurdle part and the gamma part of the model. The hurdle represented use (i.e., one) versus non-use (i.e., zero); the gamma distribution represented the time of using a medium if use was non-zero.

In total, we ran 28 models: Two considering effects based on direction (one for media use on well-being, one for well-being on media use) x two representing the well-being measure (affect and life satisfaction) x seven different forms of media. These 28 models produced 112 total parameters: 28 models x two (between vs. within) × two (use vs. time). The models directly addressed our research questions. They allowed us to estimate reciprocal effects between both use versus non-use and well-being and time spent with a medium and well-being. For each of those effects, they provided us with an estimate of the between-person relation and the within-person effect. Moreover, they estimated these effects for each medium, allowing us to assess similarities and differences in reciprocal effects between media. Last, the models provided us with an insight into whether effects will happen on short-term affect or more general evaluations of life satisfaction.

We ran all models using the *brms* package^63^ with default priors of the package. Because the data were nested, each participant received a random intercept. We also estimated random slopes for within-effects^64^ to be able to generalize within-effects to the population^65^. Note that audiobooks were so rarely used that zero inflation was extremely high, which led to poor model diagnostics. Estimates for that model are less reliable than those of the other models. For a full account of the modelling procedure and model diagnostics, see https://digital-wellbeing.github.io/cultural-consumption/analysis.html.

## Data Availability

All data, materials, and code are available on the Open Science Framework page of this project (https://osf.io/yn7sx/). Readers can find detailed documentation of data processing and analysis at https://digital-wellbeing.github.io/cultural-consumption/.
